# Amelogenin peptide analyses reveal female leadership in Copper Age Iberia (*c*. 2900–2650 BC)

**DOI:** 10.1038/s41598-023-36368-x

**Published:** 2023-07-06

**Authors:** Marta Cintas-Peña, Miriam Luciañez-Triviño, Raquel Montero Artús, Andrea Bileck, Patricia Bortel, Fabian Kanz, Katharina Rebay-Salisbury, Leonardo García Sanjuán

**Affiliations:** 1 Department of Prehistory and Archaeology, University of Seville, C/María de Padilla s/n, 41004 Seville, Spain; 2 Department of Analytical Chemistry, University of Vienna, Vienna, Austria; 3 Joint Metabolome Facility, University of Vienna, Vienna, Austria; 4 Center for Forensic Medicine, Medical University of Vienna, Vienna, Austria; 5 Department of Prehistoric and Historical Archaeology, University of Vienna, Vienna, Austria

**Keywords:** Archaeology, Peptides, Social evolution

## Abstract

Given the absence of written records, the main source of information available to analyze gender inequalities in early complex societies is the human body itself. And yet, for decades, archaeologists have struggled with the sex estimation of poorly preserved human remains. Here we present an exceptional case study that shows how ground-breaking new scientific methods may address this problem. Through the analysis of sexually dimorphic amelogenin peptides in tooth enamel, we establish that the most socially prominent person of the Iberian Copper Age (c. 3200–2200 BC) was not male, as previously thought, but female. The analysis of this woman, discovered in 2008 at Valencina, Spain, reveals that she was a leading social figure at a time where no male attained a remotely comparable social position. Only other women buried a short time after in the Montelirio *tholos*, part of the same burial area, appear to have enjoyed a similarly high social position. Our results invite to reconsider established interpretations about the political role of women at the onset of early social complexity, and question traditionally held views of the past. Furthermore, this study anticipates the changes that newly developed scientific methods may bring to prehistoric archaeology and the study of human social evolution.

## Gender archaeology: before and today

Gender archaeology emerged in the 1960s and early 1970s from feminist discontent with androcentric views on prehistory and history that overlooked women’s contributions and roles in past societies^1^. Fifty years on, a growing body of research projects, conference proceedings, monographs, and papers bear witness to the transformation of gender into a mainstream topic of archaeological research^2–11^. Although, as an analytical category, gender is a comparatively late incorporation to modern Archaeology^6^ few will deny that it has quickly expanded into a major area of interest. Multiple topics are dealt with under the rather broad conceptual umbrella of gender, including kinship and residential patterns^12,13^, the complexity and fluidity of sex-gender systems^14^, the relationship between gender and violence^15^ as well as cultural definitions of masculinity^16^, among others. However, since the earliest studies, one topic stands out: the analysis of gender inequalities.

As a cultural construct broadly based on the biological differences between men and women, gender is not always expressed in binary terms^17^. Nevertheless, the understanding of past sex-gender systems frequently rests on the identification of biological sex. Such identification, which is of crucial importance for anthropological, demographic and sociological analysis, becomes challenging when the evidence at hand is thousands of years old. The analysis of gender asymmetries is often hindered by poor preservation of human remains resulting from factors such as soil chemistry, weathering, animal scavenging, and looting. Indeed, prehistoric societies often engaged in burial practices that involved the separation, manipulation, burning and/or partial destruction of skeletal elements^18–20^. Under those conditions, the identification of the sexually dimorphic morphological traits normally used in biological anthropology (*i.e.* in the pelvis and the cranium) is often challenging, if not impossible. While genetic sex identification is an alternative, it does require the preservation of ancient DNA, which is limited in hot and dry climatic conditions.

In the last few years, however, a new scientific technique has been developed, based on the analysis of sexually dimorphic amelogenin peptides in tooth enamel by nanoflow liquid chromatography-tandem mass spectrometry^21,22^ (see Methods section for a full description of the method). This new procedure can provide highly reliable sex determinations even for poorly preserved human skeletons. The application of this technique to prehistoric human remains^23–26^ has yielded results that are likely going to significantly modify the way gender archaeology will be approached in the future. By coupling proteomics with other recently developed scientific methods, such as isotopic and aDNA analysis, which are themselves expanding rapidly, the study of prehistoric social organisation is set to change (see examples in^27–29^). The results presented here, set in Copper Age Iberia (*c.* 3200–2200 BC) demonstrates how the addition of proteomics can transform the study of prehistoric social organisation.

## Data overview: The Ivory Lady

**Figure 1:**
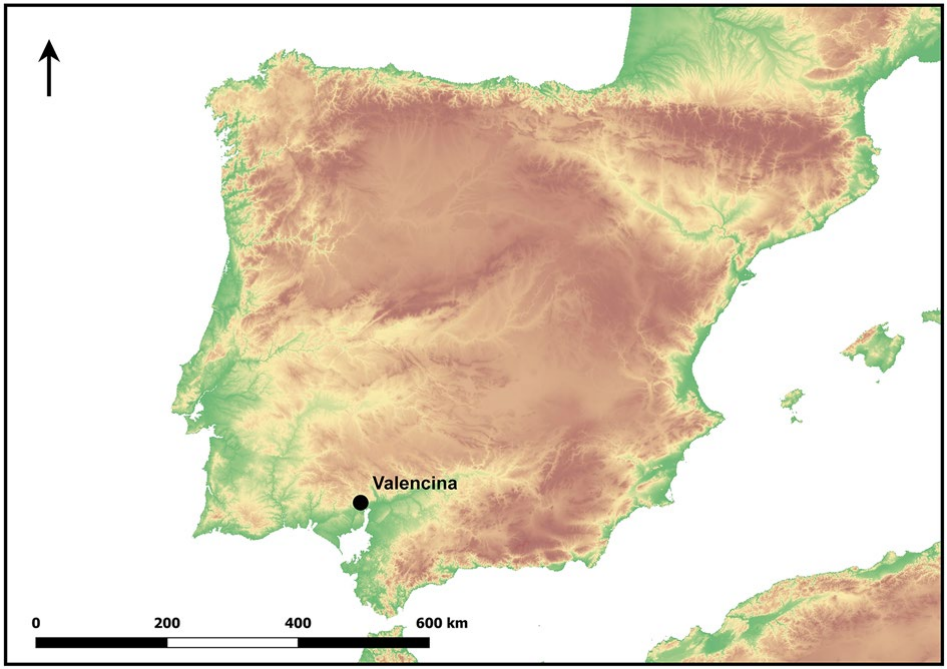
Location of Valencina. Map created using Qgis 3.22 (https://qgis.org/es/site/).

Valencina (Seville, SW Spain) (Fig. 1) is a Copper Age ‘mega-site’ spreading over *c*. 450 hectares, much larger than other coeval sites. Recent research^30^ has revealed the extent of Valencina’s monumentalism with its sophisticated megalithic chambers and massive ditches as well as its associated high-end material culture, including finely crafted sumptuary artefacts produced from exotic raw materials such as ivory, rock crystal, amber, flint, and ostrich eggshell^30^. In addition, the site has yielded the largest collection of human bone for any Iberian Copper Age site to date^31,32^. As a result, Valencina offers a unique dataset to analyse the interplay between early social complexity and gender differentiation.

**Figure 2:**
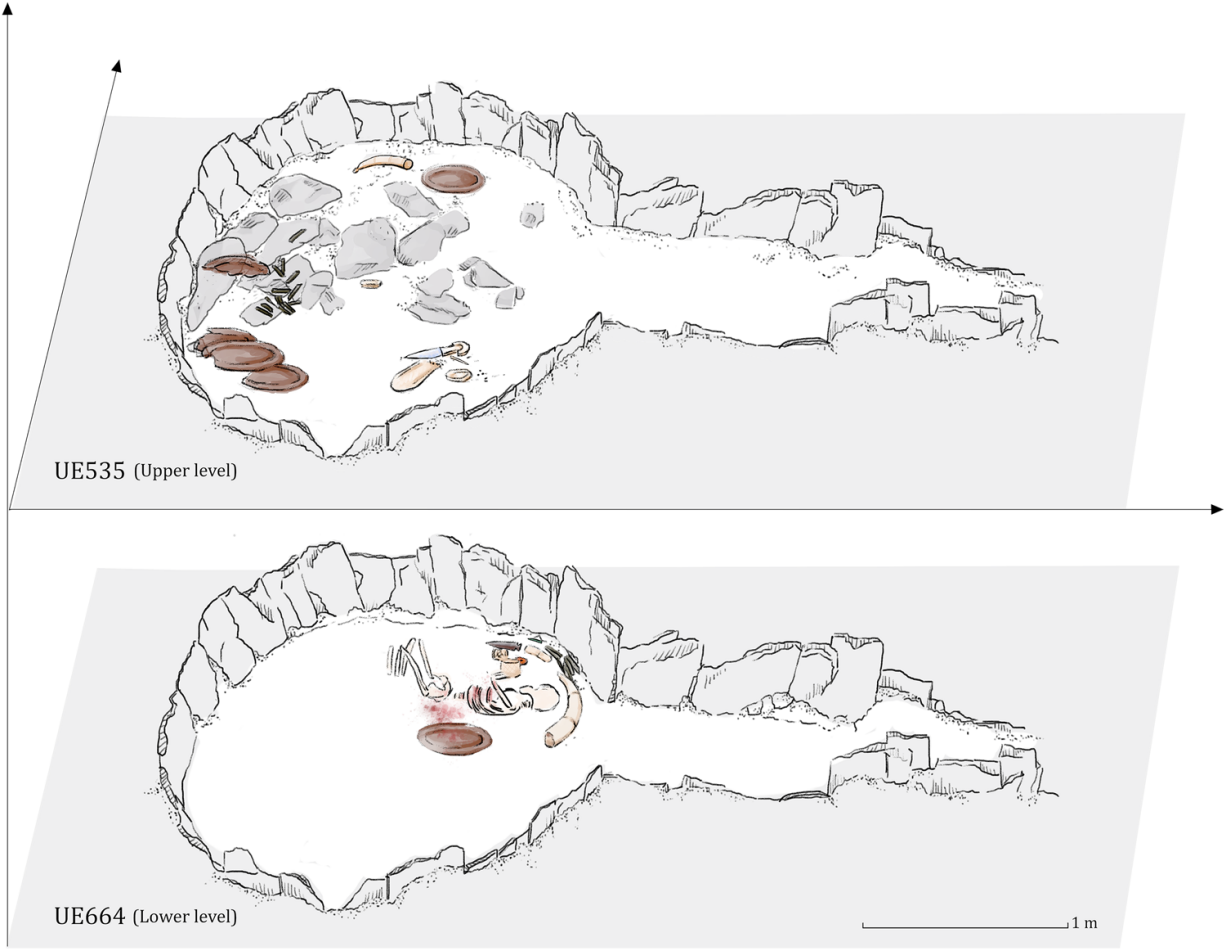
Structure 10.049, plans of the lower and upper levels. Author: Miriam Luciañez Triviño.

**Figure 3:**
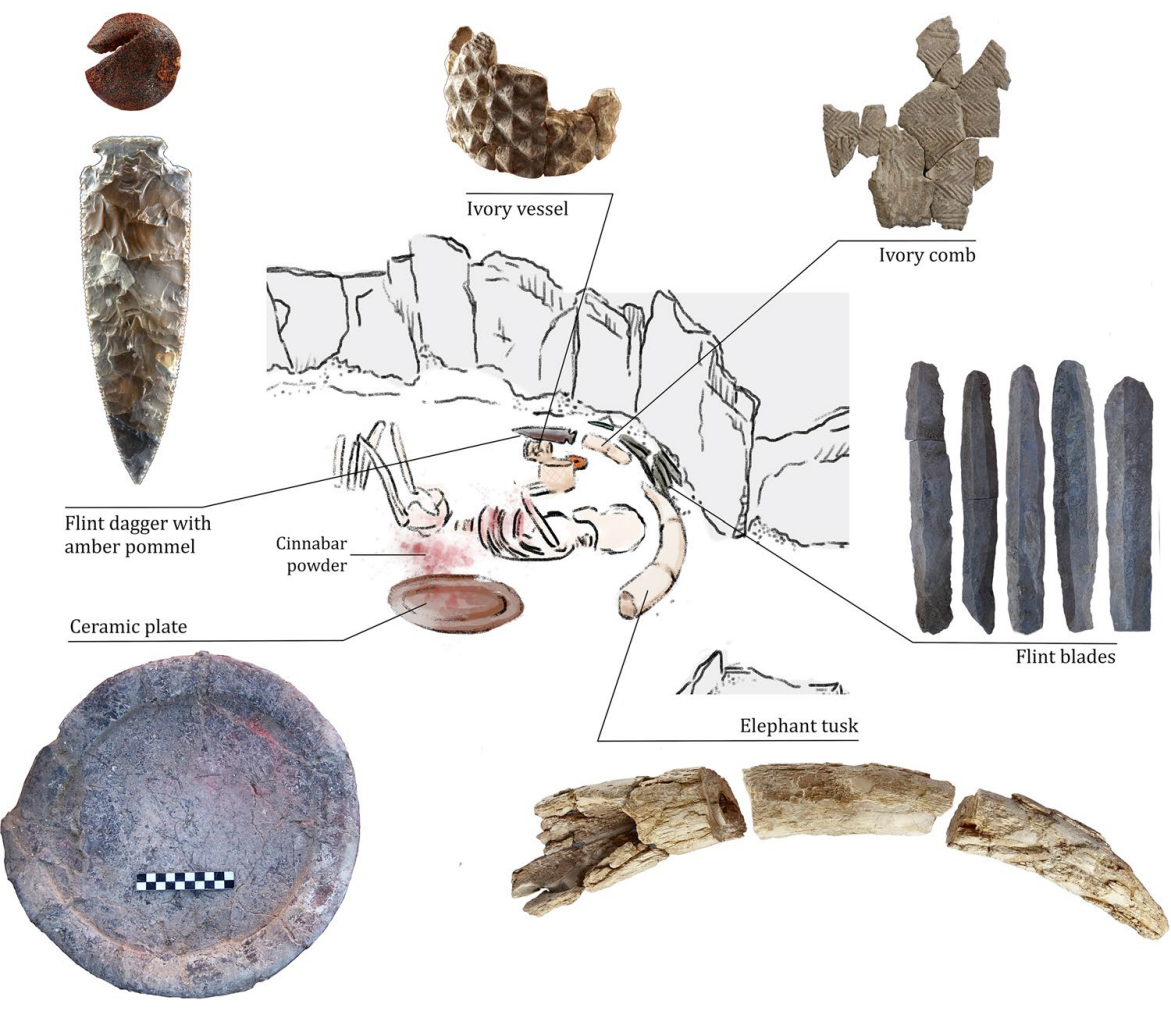
Individual buried in the lower level of the structure 10.049, and main artefacts deposited around the body. Author: Miriam Luciañez Triviño.

**Figure 4:**
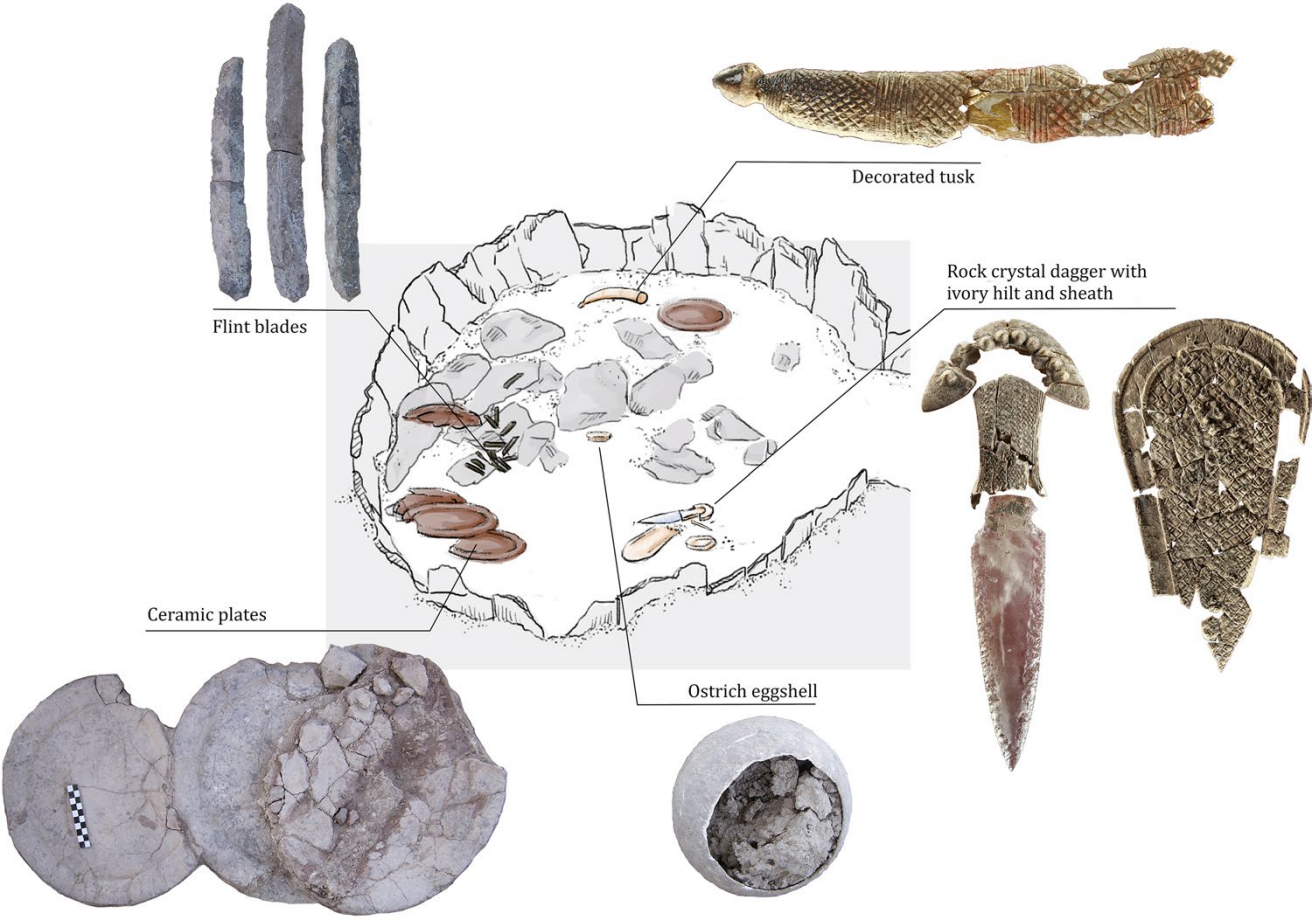
Upper level of structure 10.049, and main artefacts included in the offering. Author: Miriam Luciañez Triviño.

Even though Copper Age funerary practices in Iberia are largely characterized by collective inhumations, burial 10.049 at Valencina yielded the remains of a single individual interred as a primary inhumation (Fig. 2). This burial is remarkable for several reasons^33,34^. The individual buried in it was first identified as a probable young male between 17 and 25 years of age at the time of death based on standard anthropological analysis^35^. Strontium isotopes showed this individual to be of local origin, while at the same time, strikingly high levels of mercury in the bones revealed intense *ante-mortem* exposure to cinnabar^36^. This person was accompanied by a lavish set of prestige goods that included a large ceramic plate (in which chemical traces of wine and cannabis were found—Personal communication by Nicolas Garnier and Elisabeth Dodinet), a small copper awl and multiple flint and ivory objects (Fig. 2: lower level and Fig. 3). Remarkably, amongst the latter was a full tusk, weighing 1.8 kg, of an African elephant, which is unparalleled in western Europe^37,38^. Sometime after this burial, another offering was made to the individual buried in tomb 10.049 (Fig. 2: upper level and Fig. 4): a set of flat slate slabs were carefully laid above, and further grave goods were then deployed, including several large ceramic plates and many more ivory objects. Among the latter, a beautiful dagger with a blade made of rock crystal and an ivory handle decorated with 90 perforated discoid beads made of mother of pearl stands out^37,39^.

The quantity and quality of the artefact assemblage used as burial offering implies that this young person was the most socially prominent individual in the whole pre-Beaker Copper Age of the Iberian Peninsula (*c*. 3200 to 2500 cal BC), as a recent comparative review has shown^40^. Furthermore, the fact that none of the infant burials found in Valencina have grave goods suggests that, among the communities that lived in or frequented this site, social status was not ascribed by birth (and therefore no significant inheritance of wealth occurred), contrary to what would happen later in the Early Bronze Age)^32^. Therefore, it is fair to assume that the individual inhumed in structure 10.049 gained a prominent social position through merit and personal achievement and did not ‘inherit’ it by birth. These characteristics, together with the promotion of communal work (such as the construction and maintenance of monuments) and conspicuous consumption (feasting) match those employed by anthropologists as M. Sahlins to explain the ‘big-man’ concept^41^—but see^42^ for a critique. This concept, together with that of ‘aggrandizers’ in transegalitarian societies^43^ has been used within the context of Late Neolithic and Copper Age Iberia ^44,45^.

**Figure 5:**
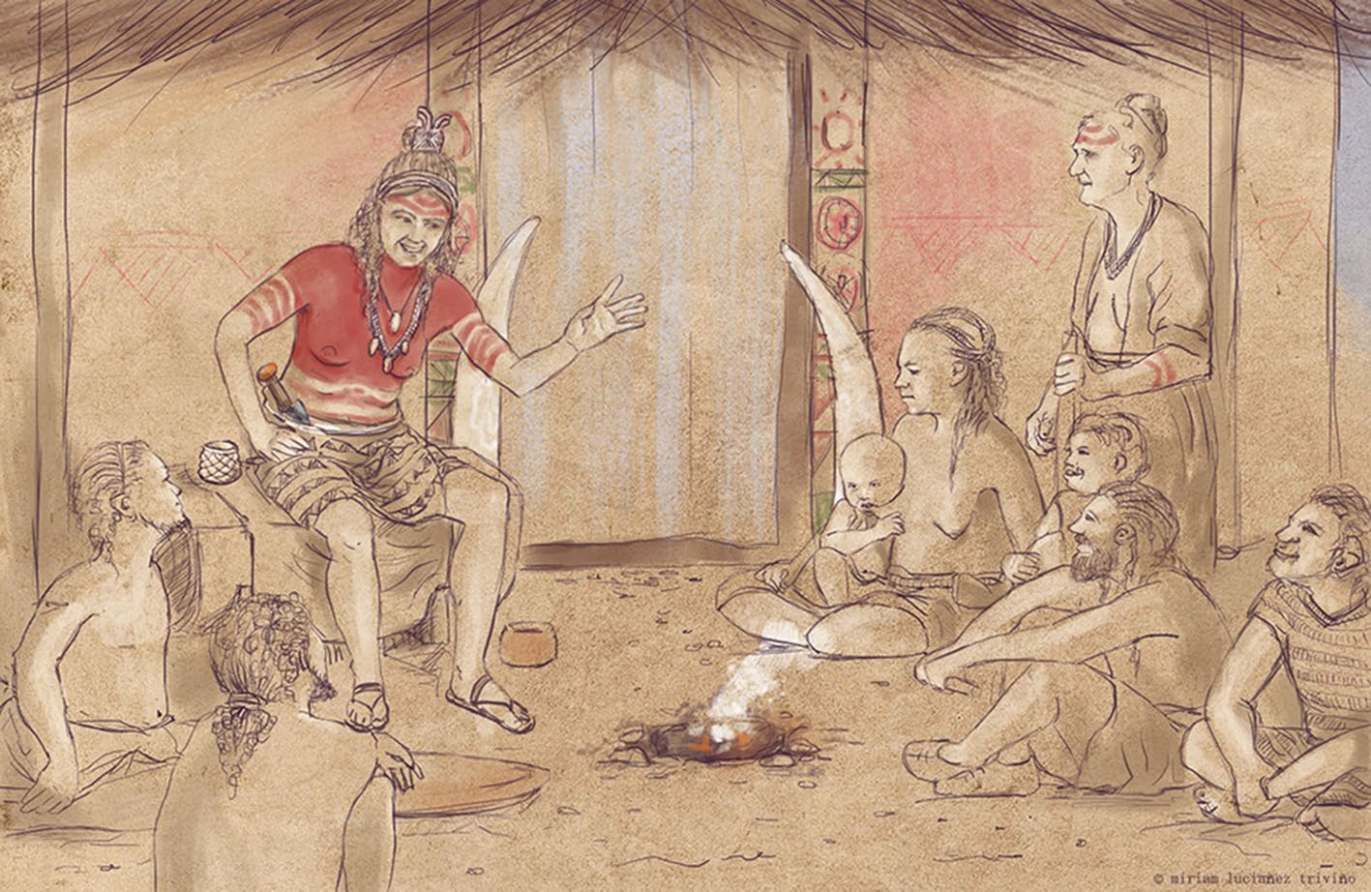
Recreation drawing of ‘The Ivory Lady’. Author: Miriam Luciañez Triviño.

In the autumn of 2021, in the scope of broader cooperation on sex and gender systems in European later prehistory^46^, peptide analysis showed that the chromosomal sex of the individual in Structure 10.049 was female, revealing ‘The ‘Ivory Man’ to be ‘The Ivory Lady” (Fig. 5).

## Copper age female leadership

This revelation foregrounds the connection of this woman with the people buried in the neighbouring Montelirio *tholos*^47^. Located barely 100 m to the south of burial 10.049, Montelirio is a two-chambered *tholos*-type monument that contained the remains of grand funerals occurring two or three generations after the “Ivory Lady”. These funerals involved 25 people, three of whom were buried in the corridor, 20 in the Large Chamber and two in the Small Chamber. A Bayesian model based on 22 radiocarbon dates on human bone suggested that the use of the Montelirio *tholos* started in 2875–2700 cal BC 2σ and ended in 2805–2635 cal BC 2σ^48^. While the remains found in the Small Chamber of Montelirio were badly disturbed in Roman times, the Large Chamber provided good-quality anthropological and artefactual data. According to the osteological study, 15 of the 20 individuals buried in it (all of them primary inhumations) were female or likely female, while the remaining five are undetermined^49^. All of them were adults at the time of death, with a predominance of ages between 20 and 35 years^49^. A major collection of high-end artefacts was recovered from the tomb, many of them manufactured from exotic raw materials, including ivory, rock crystal, gold, amber, mylonite, and flint. In addition, some of the women in the Large Chamber were dressed in sophisticated attires made with thousands of perforated beads carved from marine shell, including a full-body tunic.

Much like 'The ‘Ivory Lady’, most of the women buried in the Large Chamber of Montelirio displayed extraordinarily high levels of mercury in their bones, suggesting intense *pre-mortem* exposure to cinnabar^36,50^. In addition, one of the women presented a case of post-axial polydactyly (six toes at each foot), which probably marked her as a special person during her life, as is common in numerous cultures worldwide^51–54^. Based on their highly ‘choreographed’ burial arrangement, demographic profile, osteo-biographical characterization and associated material culture, these women were interpreted as a group of religious specialists^55^. Indeed, various pieces of evidence drawn from the analysis of the material culture found both in tomb 10.049 and in Montelirio suggest that the offerings found above ‘The Ivory Lady’ were made when Montelirio was built, two or three generations later^34^. The builders of Montelirio sought to underline the connection which tied them with ‘The Ivory Lady’. Neither in Valencina nor in the whole of the Iberian Cooper Age has any other grave been found which remotely compares in material wealth and sophistication to these two graves. These results raise entirely new questions regarding the nature of early forms of political leadership not only in Valencina but among Iberian Copper Age communities as a whole, the role played by women within them, and the specific relationship of ‘The Ivory Lady’ with the people buried in the neighbouring Montelirio *tholos*.

First, we stress that ‘The Ivory Lady’ was the person with the highest social status dating to the pre-Beaker Copper Age (*c.* 3200–2500 BC) for the whole of Iberia, based on the currently available evidence. This is significant not only because a woman distinctly appears as the most powerful person of this crucial period, when processes leading to a more hierarchical society were at play across Western Europe, but also because there is no comparable or analogous male counterpart in Iberia. The only other persons buried with comparable pomp and wealth at that time were also women: the 20 individuals, including 15 females, found in the Large Chamber of the Montelirio *tholos*. These findings are highly relevant to the study of gender asymmetries in European Late Prehistory. Well-documented cases of high-ranking Bronze Age or Iron Age women buried with substantial wealth, such as those from La Almoloya^56^, Franzhausen^57^ or the so-called ‘Princess of Vix’^58^, to name but a few, occurred within a social context in which high-standing male burials were prevalent. In comparison, the Iberian Pre-Beaker Copper Age record shows no male burials remotely comparable to those of grave 10.049 or Montelirio at Valencina.

Second, early 3^rd^ millennium Iberian societies do not display the traits indicative of highly stratified, class-like or state societies identified elsewhere in Bronze Age or Iron Age Europe. As the Valencina record shows, leadership systems in the Iberian Copper Age were at best unstable and financed by a wealth economy rather than a staple economy^59^. ‘The Ivory Lady’ appears to have drawn her influence, prestige, or power neither from birth, nor from the control of agricultural produce, but from her personal charisma and her achievements. Her association with substances such as cinnabar, including high levels of mercury exposure, wine and cannabis, are unlikely to result from exclusively mundane practices.

Third, the Valencina evidence raises further questions regarding the nature of European Late Neolithic and Copper Age societies. While there is growing evidence of gender differentiation^13^, tomb 10.049 and the Montelirio *tholos* suggests that, within the context of the incipient dynamics of social hierarchisation occurring between the late 4^th^ and early 3^rd^ millennia BC in Valencina, women ostensibly enjoyed high-ranking positions not attained by men. It is worth noting that both structure 10.049 and the Montelirio *tholos* are the most sumptuous tombs for the whole of Copper Age Iberia (and, notably, the pre-Beaker Copper Age), which suggests women held positions of leadership.

The evidence from Valencina makes a significant contribution to the wider research on gender differences and the role of women in early political organisations. Since the nineteenth century, the idea of a prehistoric past in which women were rulers has been pervasive^60–63^. The absence of decisive archaeological evidence for a matriarchy, which would support this socio-evolutionary idea, led most scholars to dismiss this approach completely^64–66^. Considering the empirical findings presented in this paper, themes such as matriarchal political systems and the role of female leaders in early political organisations deserve further discussion. The examples discussed here invite us to reconsider prevailing ideas about power, social complexity, and gender differences among early complex societies. Moreover, it opens the door to reflect on the role that nineteenth century discourse about wealth and gender play in modern interpretations, and the power of new scientific methods to challenge long-standing narratives of the past in the social sciences and the humanities.

## Methods

The recently developed method of sex determination via sexually dimorphic amelogenin peptides in human tooth enamel^22,23^ represents a breakthrough for both archaeology and anthropology. Tooth enamel contains sex chromosome-linked isoforms of amelogenin, an enamel-forming protein, which preserves well even in archaeological specimens. These sex-specific peptides can be identified by nanoflow liquid chromatography-tandem mass spectrometry (nanoLC-MS/MS). Applicable for both adult and deciduous teeth, the method is particularly useful for identifying the sex of children^25,67^, whose morphology is not yet sexually dimorphic.

In autumn 2021, the upper left first incisor (FDI 21) from individual 10.049 at Valencina was submitted to the Center for Forensic Medicine, Medical University of Vienna, Austria, for peptide-based sex identification.

The sample for peptide extraction from human tooth enamel was prepared using a previously described protocol^24,25^. In brief, a *c*. 2 × 2 mm area of the tooth enamel was first abraded using sandpaper, subsequently washed with hydrogen peroxide and rinsed with MS grade water. The abraded part was immersed in hydrochloric acid, etched for two minutes and the resulted solution was discarded; a second etch was performed and further processed for analysis. C18 ZipTips were used for the peptide clean-up procedure. After eluting the peptides with elution buffer, the sample was dried in a vacuum concentrator and reconstituted in 30% formic acid containing four synthetic standard peptides (10 fM, Supplementary Table S1) for internal quality control. 10 µL Eluent A (98% H2O, 2% ACN, 0.1% FA) were added.

A Dionex Ultimate 3000 nano LC-system coupled to a Q Exactive orbitrap mass spectrometer (Thermo Fisher Scientific) equipped with a nanospray ion source (Nanospray FlexTM, Thermo Fisher Scientific) and a stainless-steel nano bore emitter (Thermo Fisher Scientific) were employed at the Institute for Analytical Chemistry of the University of Vienna. LC–MS conditions were an adapted version of a recently published method^23^.

The software Skyline (version 20.1.0.79^68^) was employed to monitor high-quality peptide candidates in a filtering step. Isotope distribution proportions were monitored via the Skyline idotp score, with an applied cut-off of > 0.95 and a mass tolerance of 5 ppm. In order to monitor retention time shifts and confirm peptide identification, retention time differences Δt between mean AMELX/AMELY precursor ion retention times and mean retention times of closest synthetic standard peptide precursor ions were calculated. The data that meet defined quality threshold (Mass tolerance 5 ppm, Skyline idotp score > 0.95 for Enamelin and AMELX/AMELY, Δt < 4 min) were interpreted as providing reliable results.

## Results

The analysis of the upper right third molar (FDI 18) of individual 10.049 at Valencina resulted in the detection of AMELX in absence of AMELY, suggesting female as chromosomal sex. As the precursor ion intensity of AMELX was low, a second tooth, a left upper first incisive (FDI 21) was analysed, confirming female chromosomal sex of the buried individual.

The individual log peak areas of AMELX/AMELY precursor ions are shown in Supplementary Table S2, as well as their sums and the ratios of the sums between AMELY and AMELX to provide an overview of the isotope distribution proportions.

The mass spectrometry proteomics data have been deposited to the ProteomeXchange^69^, Consortium (http://proteomecentral.proteomexchange.org) via the PRIDE partner repository^70^.

## Supplementary Information

Supplementary Tables.

## Data Availability

The mass spectrometry proteomics data have been deposited to the ProteomeXchange^69^, Consortium (http://proteomecentral.proteomexchange.org) via the PRIDE partner repository^70^ with the dataset identifier PXD038664 and 10.6019/PXD038664.
