# Supplementary material for: Amelogenin peptide analyses reveal female leadership in Copper Age Iberia (c. 2900–2650 BC)
Source: Sci Rep. 2023 Jul 6;13:9594. doi: 10.1038/s41598-023-36368-x (PMC10326254; doi:10.1038/s41598-023-36368-x)
Supplement: Supplementary file 1 — Supplementary Tables. [file 41598_2023_36368_MOESM1_ESM.pdf]

Peptide name	Sequence	MH+	Provider
Glu1-Fribrinopeptide B	EGVNDNEEGFFSAR	1570.67684	Sigma F3261
M28	TTPAVLDSDGSYFLYSK	1863.90109	PSL
HK0	VLETKSLYVR	1207.70504	PSL
HK1	VLETK(ε-AC)SLYVR	1249.71561	PSL

Table S1. Specifications of the synthetic standard peptides added for internal quality control.

AnChem ID	Grave/ tooth	AMELX (750.7368 m/z)					AMELY (483.7393 m/z)					Sum log Peak Areas AMELX / Sum log Peak Areas AMELY	Peptide predicted sex	Archeological gender assessment
		Log Peak Areas				Δt [min] to STD 932.4542++	Log Peak Areas				Δt [min] to STD 604.3559++			
		M	[M+1]	[M+2]	Sum		M	[M+1]	[M+2]	Sum				
21-PEP-2532	10049/ FDI=21	8,04	8,20	8,11	24,35	2,72	-	-	-	-	-	-	Female	Male?
22-PEP-0435	10.049/ FDI=18	6,81	7,03	6,90	20,75	2,49	-	-	-	-	-	-	Female	Male?

Table S2. The individual measurement data including log peak areas of AMELX/AMELY precursor ions, as well as their sums and resulting sex prediction.
